# The history behind successful uterine transplantation in humans

**DOI:** 10.5935/1518-0557.20170028

**Published:** 2017

**Authors:** Luis Arturo Ruvalcaba Castellón, Martha Isolina García Amador, Roberto Enrique Díaz González, Montoya Sarmiento Jorge Eduardo, César Díaz-García, Niclas Kvarnström, Mats Bränström

**Affiliations:** 1 Mexican Infertility Institute [Instituto Mexicano de Infertilidad (imi)], Guadalajara, Jalisco-Mexico; 2 Department of Gynecology and Obstetrics, La Fe University Hospital, University of Valencia, Valencia, Spain; 3 Sahlgrenska Academy at University of Gothenburg, Gothenburg, Sweden; 4 Stockholm IVF, Stockholm, Sweden

**Keywords:** infertility, transplantation, uterus

## Abstract

This paper aimed to describe the basic aspects of uterine transplant (UTx) research in humans, including preliminary experiences in rodents and domestic species. Studies in rats, domestic species, and non-human primates validated and optimized the UTx procedure in terms of its surgical aspects, immunosuppression, rejection diagnosis, peculiarities of pregnancy in immunosuppressed patients, and patients with special uterine conditions. In animal species, the first live birth from UTx was achieved in a syngeneic mouse model in 2003. Twenty-five UTx procedures have been performed in humans. The first two cases were unsuccessful, but established the need for rigorous research to improve success rates. As a result of a controlled clinical study under a strictly designed research protocol, nine subsequent UTx procedures have resulted in six healthy live births, the first of them in 2014. Further failed UTx procedures have been performed in China, Czech Republic, Brazil, Germany, and the United States, most of which using living donors. Albeit still an experimental procedure in, UTx is the first potential alternative for the treatment of absolute uterine factor infertility (AUFI).

## INTRODUCTION

The clinical field of tissue transplantation now includes uterine transplants (UTx). UTx has its peculiarities. It is a transitory procedure that does not necessarily save a life in danger, but instead improves quality-of-life and offers women anatomically or functionally unable to bear children the possibility of becoming mothers and giving birth to healthy infants. Hysterectomy is indicated after parity mainly to avoid the risks secondary to long-term immunosuppression.

Absolute uterine factor infertility (AUFI) affects approximately one in 500 women of childbearing age (Milliez, 2009), or 1.5 million women worldwide. Motherhood options traditionally offered to women with AUFI revolve around adoption or surrogacy. Adoption only provides legal motherhood, while a surrogacy agreement confers genetic motherhood and, after adoption, legal motherhood to infertile mothers. During a process of this kind, multiple medical and legal issues may arise. Maternal surrogacy is prohibited in many countries, including France, Germany, Bulgaria, Croatia, Estonia, Finland, Hungary, Italy, Lithuania, Portugal, Slovakia, Australia, Holland, Spain, Sweden, and Norway, while in others there is no legislation or regulation (Laurence *et al.*, 2012) on the matter, which often leads to conflict between the involved parties.

UTx emulates a normal situation with the primary components of maternity: genetic, gestational, and legal. Unlike individuals opting for surrogacy, women given a uterine graft assume health risks associated to transplantation, pregnancy complications, and immunosuppression. The duration of the allograft will essentially be determined by the possibility of pregnancy and parity satisfaction in the recipient. Once parity has been achieved, the uterus is surgically removed to allow the suspension of immunosuppressants, thus minimizing long-term side effects.

The UTx clinical trial that has achieved the greatest number of live births followed the principles of the IDEAL (Idea-Development-Exploration-Assessment-Long-term study) framework (McCulloch *et al.*, 2009). This model places emphasis on the importance of preclinical research to structure improved surgical procedures and on the discussion and prior approval of the protocol by local and/or national ethics committees.

This paper aimed to review and summarize historical aspects related to successful UTx in humans.

### Uterine absence: congenital or acquired

#### Congenital absence of the uterus

The uterus is formed in the early stages of fetal life through the fusion of the Müllerian ducts. Müllerian duct agenesis occurs in one of every 4,000 to 10,000 women. Its most frequent clinical manifestation is utero-vaginal agenesis, or Mayer-Rokitansky-Küster-Hauser (MRKH) syndrome, a rare congenital condition that significantly impacts the lives of affected women. The most common phenotypes of this congenital disease are the absence of the vaginal canal, with a narrow vaginal introitus, absent uterus or rudimentary uterine horns, and reduced or absent uterine cavity, with structurally and functionally normal ovaries (Grimbizis *et al.*, 2001). In most cases, surgery focuses on the creation of a neovagina through various methods to partially restore sexual function. However, in no way does this treatment address or resolve the reproductive needs of patients. Individuals with other anomalies in the development and fusion of the Müllerian ducts, such as hypoplastic uterus, cannot benefit from surgery and suffer from high failed implantation and miscarriage rates (ACOG, 2013).

#### Acquired anatomical or functional absence of the uterus

Leiomyomatosis is probably the most common surgical cause of hysterectomy (Marshall *et al.*, 1997), with an incidence in women of reproductive age as high as 10% (Borgfeldt & Andolf, 2000). Multiple and/or submucosal myomas cause failed implantation or hysterectomy, depending on their number, size, and location. Other causes of surgical resection of the uterus are oncological surgery for cervical or endometrial cancer and emergency procedures for obstetrical complications, including massive hemorrhage due to uterine rupture, atony, and/or placenta accreta.

Asherman syndrome (dense intrauterine adhesions) has been associated with symptoms such as alterations in menstrual patterns, including amenorrhea, hypomenorrhea, recurring miscarriage, and abnormal placentation in cases of previous pregnancies. In most cases, this condition has been associated to endometritis or surgical abortions and is also known as one of the main causes of AUFI. However, in many cases, despite attempted hysteroscopic treatment, approximately 50% of women are left infertile (Fernandez *et al.*, 2006).

The first scientific publications on UTx date back to the 1960s, when combined uterine-oviduct transplant was tested in dogs (Eraslan *et al.*, 1966). At that time, the main objective was to find a treatment for the many women who were infertile due to tubal obstruction secondary to inflammatory rather than surgical processes. The results were not good, likely due to the poor efficacy of the available immunosuppressant drugs available at the time.

After the birth of Louise Brown in 1978, *in vitro* fertilization (IVF) was clinically approved and became a highly effective treatment regardless of the factors involved in the etiology of infertility, as it was also effective for cases of tubal origin. Consequently, research into uterine-oviduct transplant ended.

Brännström *et al.* began the UTx research project in 1999, including experiments in a variety of animal species. These studies will be summarized later (Díaz-García *et al.*, 2012).

### Research in animal models

#### Mouse

In 2002, pregnancy was achieved in a transplanted mouse (El-Akouri *et al.*, 2002; El-Akouri *et al.*, 2003). The offspring had normal post-natal growth patterns and were fertile. The influence of immunosuppression was not demonstrated in this group, as the transplants were between animals of the same endogamic strain (syngeneic), with no need for immunosuppression. Experiments in this animal model showed normal pregnancy and in utero development of pups in uteruses that had undergone cold ischemia for 24 hours prior to transplantation (El-Akouri *et al.*, 2003). Rejection was characterized in allogeneic UTx mouse models (El-Akouri *et al.*, 2006; Groth *et al.*, 2009). More specifically, the authors showed that cyclosporine monotherapy overcame graft rejection (Wranning *et al.*, 2007) and that exposure to this drug during pregnancy affected reproductive capacity by decreasing implantation and fetal survival rates (Groth *et al.*, 2010).

#### Rat

UTx in rats developed later as a model more comparable to the human model as it concerns therapeutic levels of immunosuppressant drugs. The uterus was transplanted in an orthotopic position, with vascular anastomoses to the common iliacs (Wranning *et al.*, 2008). Spontaneous conception and successful pregnancies after UTx were achieved in a syngeneic rat model (Wranning *et al.*, 2011). In allogeneic UTx models, tacrolimus (Akhi *et al.*, 2013) was more effective than cyclosporine (Groth *et al.*, 2012) in preventing rejection. As a result, tacrolimus was used in subsequent experiments (Díaz-García *et al.*, 2010). Other experiments continued to demonstrate normal pup development in subjects submitted to UTx on immunosuppression (Díaz-García *et al.*, 2014) and revealed that the uterus is highly resistant to warm ischemia (Díaz-García *et al.*, 2013).

#### Rabbit

Smith *et al.* in London explored the rabbit UTx model. In their initial protocol, the authors used the aorto-caval vascular patch technique in an allogeneic model on immunosuppression with tacrolimus (Saso *et al.*, 2014). Two of five animals survived the postoperative period, but only one had a lasting uterus, although it was very small in size as revealed in the autopsy performed ten months after transplantation. In one follow-up study that included new UTx procedures, only one rabbit survived for more than a month, showing the greater difficulty of this surgical procedure in this animal species (Saso *et al.*, 2014). Three episodes of rejection in this group were controlled with prednisolone and a temporary doubling of the dosage of tacrolimus. After embryo transfer, one early pregnancy was observed on ultrasound examination, but it ended in a miscarriage. Thus, no live births after UTx have been recorded in rabbits.

#### Sheep

The advantages of performing UTx in sheep revolve around the similarities in size and anatomy of the pelvic vasculature they bear with humans. Initially, an ovine auto-UTx model was developed (Dahm-Kahler *et al.*, 2008), with the excision of a uterine horn and the retrieval of a wide unilateral vascular pedicle, including the anterior ramus of the internal iliac artery and the complete utero-ovarian vein. Next, and end-to-side anastomosis was performed on the external iliacs. In this model, the reperfusion events following ischemic preservation were also described (Wranning *et al.*, 2008). In subsequent experiments, live births were achieved after auto-transplant (Wranning *et al.*, 2010). In particular, the graft was submitted to a prolonged three-hour period of warm ischemia, demonstrating that a uterus with a size similar to a human uterus could tolerate the cell changes induced by prolonged warm ischemia.

A group led by Ramirez in Colombia used an allogeneic UTx model in sheep to demonstrate the long-term survival of the uterus using immunosuppression with cyclosporine (Ramirez *et al.*, 2008). This finding was followed by the first live birth following allo-UTx in a large animal (Ramirez *et al.*, 2011). In this study, three of every 12 sheep achieved pregnancy after embryo transfer and had live births. The two studies (Ramirez *et al.*, 2008; Ramirez *et al.*, 2011) used a surgical protocol with end-to-end anastomosis of the uterine vessels.

This type of anastomosis between uterine vessels can only be performed when the recipient undergoes a hysterectomy at the time of transplantation, which may be relevant to humans in the rare cases of AUFI caused by uterine adhesions or uterine malformations. The same type of anastomosis was used by a group in China, who reported graft survival of one month after allo-UTx with immunosuppression induction, followed by a tripleimmunosuppressant scheme (steroids, azathioprine, and/or cyclosporine/tacrolimus) (Wei *et al.*, 2013). In contrast, Tzakis *et al.* described an approach for the vaginal insertion of the heterotopic UTx with a cutaneous stoma and vascular anastomoses to the aorta and vena cava (Gonzalez-Pinto *et al.*, 2013).

#### Pig

Pigs have also been used as a large-animal model in UTx research. Venous effluents from autologous UTx have been examined (Wranning *et al.*, 2006) for blood gases, lactate, and thiobarbituric acid reactive species (TBARS), the latter used as an indicator of oxidative stress. Blood gases and lactate concentrations normalized after approximately 60 min, and TBARS levels were unchanged from those observed prior to transplantation. Smith *et al.*, in long-term studies of UTx, described the development of thrombosis in the distal end of the uterine-vessel anastomosis lines (Sieunarine *et al.*, 2005), and specifically noted the susceptibility of anastomoses of the smaller vessels. In another experimental event in allogeneic UTx in pigs, the uterus was located in a heterotopic position (Avison *et al.*, 2009). Initial immunosuppression was performed with IV tacrolimus followed by maintenance therapy with oral cyclosporine. A survival rate of 50% was reported over 12 months of follow-up. Increasing dosages of immunosuppressant drugs used for maintenance, including corticosteroids, effectively reversed acute rejection episodes.

#### Non-human primates

UTx research in non-human primates was initiated with a vascular anastomosis protocol in autologous UTx in baboons. In the initial study, menstruation was reestablished in only 20% of the subjects (Enskog *et al.*, 2010). The method was modified in relation to irrigation of the graft and anastomosis, resulting in a 3-fold higher success rate (Johannesson *et al.*, 2012). Still, no pregnancies were produced, despite attempts at breeding over a period of several months. Failure to achieve pregnancy was due to tubal obstruction, caused possibly by ischemic damage. In addition, an allogeneic UTx was performed in a baboon using a living donor and anastomosis to the external iliac vessels (Johannesson *et al.*, 2013). The organ-retrieval surgery lasted approximately three hours, and the donor-survival rate was 100%. In this study, various immunosuppression protocols were tested, and induction therapy with anti-thymocyte globulin followed by a triple immunosuppression scheme with tacrolimus, mycophenolate mofetil (MMF), and corticosteroids resulted in graft survival at three months (Tryphonopoulos *et al.*, 2014). The initial protocol for immunosuppression induction included a triple-drug regimen for some months, followed by tacrolimus only. Although graft rejection was observed, the cases were successfully treated, and graft survival exceeded 12 months.

In the small cynomolgus macaque, a Japanese research team performed autologous UTx experiments spending 6-8 hours in the organ-retrieval surgery and 4-6 hours in the anastomosis surgery (Kisu *et al.*, 2012). This experiment produced the first pregnancy after any type of UTx in a non-human primate species (Mihara *et al.*, 2012). The animal underwent auto-UTx, involving bilateral anastomosis of the uterine artery and vein to the external iliac arteries. Natural mating caused pregnancy, which developed normally until the occurrence of placental abruption close to term. A direct offspring was achieved. In a follow-up study on allogeneic UTx in this species, resumption of menstruation was seen in an animal under immunosuppression with tacrolimus, methylprednisolone, and mycophenolate mofetil (Kisu *et al.*, 2014).

#### Uterine transplant in humans

In the year 2000, in Jeddah, Saudi Arabia, the first salpingo-UTx was attempted using a living donor in a woman submitted to emergency peripartum hysterectomy. Although the transplant did not result in pregnancy, it is credited with having achieved living-donor and recipient surgeries without major complications. However, the uterus in the recipient only remained viable for 100 days (Fageeh *et al.*, 2002); the donor had a perioperative ureteral lesion, and it is questionable whether the uterus was correctly perfused. A necrotic uterus was removed after three months (Díaz-García *et al.*, 2012).

In 2011, a second human UTx was attempted. In Antalya, Turkey, a 21-year-old patient with MRKH received a uterus from a deceased 22-year-old donor (Akar *et al.*, 2013; Ozkan *et al.*, 2013). The donor's multi-organ retrieval surgery lasted two hours, and the uterus was the first organ to be procured. The transplant procedure lasted six hours and included bilateral end-to-side anastomosis of the graft common iliac vessels to the external iliac vessels. The immunosuppression protocol included thymoglobulin for ten days and maintenance suppressive therapy with a triple-drug regimen with prednisolone, mycophenolate mofetil, and tacrolimus. Eighteen months after UTx, the embryo-transfer attempts began. The patient attempted IVF multiple times, but only two very early miscarriages were observed (Akar *et al.*, 2013). The reasons for the failed pregnancies are unknown in this case; however, it is important to bear in mind that a nulliparous uterus was transplanted and that its capacity to carry a pregnancy to term had not been demonstrated.

#### Organ retrieval from deceased donors

The first studies looked into uterus retrieval techniques in brain-dead donors. In the initial study, some 150 multiorgan acquisitions were identified as possible procedures for the donation of a uterus for research purposes (Del Priore *et al.*, 2007). Donation of the uterus was accepted in only six percent of the cases. The research protocol for the procurement of uteruses was designed to ensure the harvesting of complete internal iliac vessels, but in most cases only the proximal portions were harvested. In a later study performed in France with the same purpose, seven uteruses were collected; consent was received in fifty percent of the cases (Gauthier *et al.*, 2014). The uteruses were collected after the procurement of thoracic and abdominal organs. In situ perfusion of the organs was performed with catheters placed in the femoral arteries. The internal iliac arteries and veins were successfully preserved bilaterally in six of seven cases. It was concluded that uterus retrieval could be part of a multiorgan procurement procedure with reproducible results. Retrieval of the organ from a deceased donor allows a wide dissection of the vascular pedicles and, as a result, wider vascular anastomoses, minimizing the risk of thrombosis and increasing the likelihood of prompt re-establishment of blood flow to the transplanted organ. Furthermore, the ureteral section (closely linked to the uterine vessels), with which dissection of the ureteral tunnel is avoided, decreases total surgery time.

The consistent and successful UTx protocol of Brännström *et al.* (2014) was determined as follows:

#### Recipient selection

A preliminary review process was conducted with a total of 30 candidates, from which ten were selected. During the selection process, an additional candidate was excluded from the project for presenting bilateral pelvic kidneys.

In the framework of this translational research, nine UTx procedures were performed. Eight were carried out on patients with MRKH (congenital uterine absence) and one on an individual with a history of hysterectomy for cervical cancer. Ovarian stimulation, oocyte aspiration, and embryo cryopreservation were performed prior to UTx, since the transfer of embryos would be performed at least one year after transplantation, as recommended by international guidelines (Brännström *et al.*, 2014).

Recipients and their respective partners were informed and advised of the possibility of satisfying the need for having a child through adoption or surrogacy, and signed the pertinent consent forms prior to hysterectomy and transplantation.

#### Donor selection

The majority of donors were close relatives or family members of the recipients. Five of them were postmenopausal donors. All had more than one pregnancy and normal live birth in their reproductive history. The post-menopausal donors received cyclical hormone therapy for a few months before uterus removal until a normal menstrual pattern was achieved. General data on the recipients and donors and aspects related to the UTx procedure are reviewed in detail in Box 1. Donors and recipients were evaluated by a multidisciplinary team of specialists and with the aid of immunological, radiological, hematological, microbiological, and hepatorrenal function diagnostic tests.

#### Surgery in living donors

In order to explore the length of the vascular pedicles with a living uterine donor, a study was performed with the additional step of a dissection of the uterine vein besides the normal dissection of the uterine artery during radical hysterectomy for cervical cancer. The additional dissection of the uterine veins added approximately 30 minutes to the three to four hours of the original procedure, but did not affect postsurgical morbidity (Johannesson *et al.*, 2012).

Procurement of the surgical specimen from the donor involved the isolation of the uterus with long bilateral vascular pedicles, including the internal iliac arteries distal to the emergence of the superior gluteal artery and the uterine veins from below the internal iliac veins (Brännström *et al.*, 2014). In order to guarantee adequate fixation of the uterus in the recipient, parts of the round and utero-sacral ligaments were preserved in the living donor, and a broad resection of the vesicoperitoneum was performed. Salpingectomy was performed with the basic goal of preserving the uterine branches of the utero-ovarian veins. In the pelvic wall, a bilateral dissection of the ureters was performed at their point of bifurcation from the iliac vessels distal to their entrance to the bladder, on par with a meticulous dissection of the uterine vessels from their close proximity adhering to the ureters. After completing the dissection and with the complete mobilization of the ureters and their separation from the uterine vessels and cervix, vascular dissection of the internal iliac arteries and veins was completed from their bifurcation. The vagina was sectioned 1-1.5 cm from the base of the vaginal cul-de-sac. A suprapubic drainage catheter was placed and remained for 4-5 days until a residual urine volume < 150 mL was obtained. Surgery in donors lasted an average of ten hours. Perioperative outcomes were favorable in all cases, with an inpatient stay of six days. In one patient, a ureterovaginal fistula was diagnosed two weeks later and was repaired.

#### Pre-transplant organ preparation

One of the most delicate aspects of the transplant process is the cryopreservation of the organ prior to collection, since much of its post-transplant function relies on successful preservation (Wranning *et al.*, 2005).

Initially, the surgical specimen was washed with heparinized saline solution and then with preservation solution (Custodiol HTK solution; Nordmedica) until the solutions were completely clear. The uterus was then maintained in cold ischemia divided into three periods:

1. Warm ischemia: from clamp placement on the internal iliac vessels and retrieval of the organ until the start of organ washing in cryopreservative, for approximately two minutes;
2. Cold ischemia: total washing time until the organ was placed on ice; and
3. Warm ischemia: a second period beginning from the removal of the organ from ice to organ reperfusion.

#### Surgery in recipients

**Figure 1:**
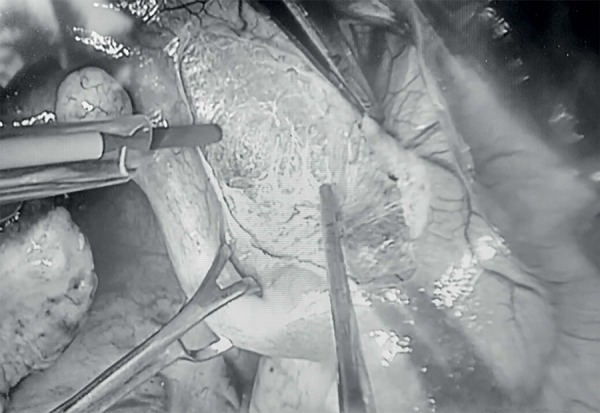
Uterine rudiment (MRKH).

**Figure 2:**
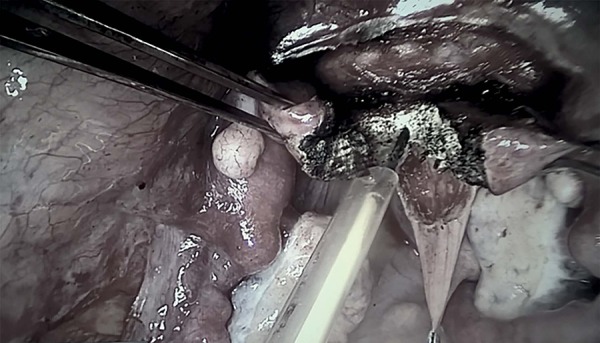
Incision in the middle of the uterine rudiment.

Preparation of tissue in the recipients was initiated in a contiguous operating room through laparotomy performed by a second team of surgeons. In the recipient, a cavity was opened through an infraumbilical and medial suprapubic incision. First, in the pelvic cavity, the base of the vaginal sac was incised to release the bladder and the rectum. In patients with MRKH (Fernandez *et al.*, 2006), the uterine rudiment (Fig.1) was incised to allow access to the vaginal cupola (Fig. 2). Subsequently, the surgery focused on the preparation of the iliac vessels. The arteries and veins were separated bilaterally from each other and the adjacent tissues over a length of approximately 6 cm. The uterus, still on ice, was brought to the operating room and placed into its normal position using bilateral end-to-side anastomoses to the external iliac vessels with 7-0 (arteries) and 8-0 (veins) polypropylene sutures. Upon completion of the anastomoses, the vascular clamp on the iliac veins was opened; in some cases, simple sutures in the anastomosis lines were necessary to seal leaks.

In six patients, the uterine branches of the utero-ovarian veins were anastomosed to the ipsilateral uterine vein or were prepared for direct anastomosis to the external iliac vein. Mannitol (30g) was administered (Mannitol Baxter Viaflo; Baxter Medical) via intravenous bolus injection just before clamp removal, and blood pressure was monitored and maintained above 100mmHg. With the vascular anastomosis completed, adequate uterine artery flow was verified with Doppler flowmetrics. Finally, a longitudinal incision of approximately 4cm at the base of the sack of the recipient was performed for the piece anastomosis in the vaginal impeller with continuous absorbable 2-0 suture. The remainder of the organ fixation process was performed with 1-0 polypropylene suture, including the round and utero-sacral ligaments and the two lateralized parts of the uterine rudiment in MRKH. The surgery lasted approximately 4-6 hours. Post-surgical hospital convalescence was 3-9 days. The most relevant aspects of the series (recipient and donor) of the uterus transplant group of Brännström *et al.* (2014) are described on Table 1.

**Table 1:** General data from recipients and donors, including data related to UTx

Pair	Age	Anesthetic Time h/min	Surgery Time h/min	Warm Ischemia h/min	Cold Ischemia h/min	Anastomosis Time (veins) min	Anastomosis Time (artery) min	Time of hospitalization after surgery
R1	33	15/0	4/10	1/18	1/30	39	35	8
D1	52	12/4	10/54					6
R2	38	13/57	4/17	1/38	1/47	43	29	9
D2	58	13/46	12/37					6
R3	28	10/50	4/50	1/34	1/4	31	25	6
D3	54	13/37	12/53					6
R4	27	6/5	5/4	1/17	0/57	32	30	6
D4	50	11/11	10/34					6
R5	35	5/45	4/55	1/13	1/6	32	30	6
D5	61	11/6	10/17					6
R6	27	8/17	4/30	1/24	2/0	60	23	3
D6	53	11/50	10/52					6
R7	28	6/35	4/44	1/15	0/54	20	30	7
D7	50	11/35	10/17					6
R8	33	7/53	5/56	1/14	0/56	25	24	8
D8	37	11/55	11/23					6
R9	35	8/14	4/31	1/32	1/28	42	47	7
D9	62	14/5	13/8					6
Recipients	31.5±3.9	9/4±3/14	4/46±0/30	1/23±1/9	1/18±0/23	36±11	30±6.9	6.7±1.6
Donors	53±7	12/13±0/60	11/37±0/2-3					6.0±

Adapted fromBrännstrom *et al*., 2014.

#### Immunosuppression

Initial immunosuppression was achieved with perioperative administration of thymoglobulin and methylpred-nisolone, followed by four days of oral glucocorticoids. The maintenance double-agent immunosuppression scheme (tacrolimus and mycophenolate mofetil) was initiated after surgery and was maintained for the first six months. The objective was to only give tacrolimus after six months, but if more than one episode of acute rejection occurred in the first six months, the initial mycophenolate mofetil (MMF) would be replaced with azathioprine with or without oral glucocorticoids. At six months of follow-up (Brännström *et al.*, 2014), seven uteruses remained in good condition, with re-establishment of regular menstruation between one and two months after UTx. Two uterine grafts were removed within the first four months, one due to bilateral uterine-artery thrombotic occlusion three days after transplantation in a recipient heterozygous for Leiden mutation.

The second hysterectomy was performed on a recipient who returned to the hospital one month after UTx for fever and vaginal secretion. An intrauterine *Enterococcus faecalis* infection was diagnosed. Despite the administration of broad-spectrum antibiotics and various attempts at surgical drainage, a persistent intrauterine abscess developed. After two months of antibiotic therapy, initial signs of septicemia were manifested, and the uterus, which was exhibiting necrotic areas, was removed 3.5 months after transplantation.

#### Transplant rejection

Mild rejection episodes, although subclinical, were diagnosed with a protocol of cervical biopsies in five of the seven patients with viable grafts. Rejection episodes were effectively reversed after two weeks of treatment with corticosteroids or increased dosage of tacrolimus. Blood flow in the uterine artery was maintained within normal limits through the first year post-transplant (Johannesson *et al.*, 2015).

#### Psychological aspects

A psychologist should evaluate donors and recipients for mental competence and emotional stability before starting the UTx protocol, since it entails major surgery on a healthy individual to help another. The emotional statuses of the recipient and the donor, regardless of kinship, may influence the process and, especially, the outcome of the procedure.

UTx is a temporary process in which the recipient undergoes a process of adapting to a received uterus, followed by treatments to achieve pregnancy through IVF, which ultimately bring a child into the world in the shortest time possible to allow the removal of the uterus. The recipient has to go through a process that brings with it a new phase of understanding, pain over the loss, and re-adaptation.

In the first year after transplantation recipients were generally optimistic, with slight levels of anxiety and stress, but great expectations over the outcome of the procedure (Jarvholm *et al.*, 2015).

#### Embryo transfer and results

Attempts to achieve pregnancy began 12 months after transplantation, using single embryo transfers. The first human live birth after UTx in this patient cohort occurred in early September 2014 (Brännström *et al.*, 2015). The recipient had an uncomplicated pregnancy up to 31.5 weeks of gestation, at which time she sought consultation due to a headache. She was diagnosed with preeclampsia, and was offered a cesarean section the next day due to anomalous fetal cardiotocography findings. The first healthy boy was born weighing 1775g and a normal size. The recipient had unilateral renal agenesis (Brännström *et al.*, 2015). To date, three other recipients have had gestational hypertensive syndrome, both with unilateral renal agenesis, and two patients presented with intrahepatic cholestasis. After the first birth, the subsequent birth weights have been greater than 2500g. One of the recipients reached the end of a second pregnancy. To date, five healthy newborns have been recorded, and one pregnancy is in progress (unpublished data, reported by Brännström, 2017). Table 2.

**Table 2:** Obstetric and perinatal outcomes of patients submitted to uterine transplantation

Diagnosis	Pregnancy Week	Weight	Indication	Apgar Score
MRKH (single kidney)	31+5	1775g	Preeclampsia	9-10-10
MRKH	34+4	2510g	Cholestasis	9-10-10
Cervical cancer	35+0 Delivered 2^nd^ child	2700g	Elective C-section	8-8-8
MRKH (single kidney)	34+5	3074g	Preeclampsia Cholestasis PPROM	3-7-10
MRKH (single kidney)	35+3 Ongoing pregnancy of 2^nd^ child	2552g	Preeclampsia	9-10-10

## CONCLUSION

UTx has demonstrated its potential as a highly effective treatment for infertility due to congenital or acquired uterine absence, especially in patients with MRKH. Other prospective observational studies must be performed to reinforce the findings, concepts, and differentiated experiences related to living and deceased donors, to support the present results, and to demonstrate their reproducibility.
